# Late-Onset Penile Fracture After Collagenase *Clostridium histolyticum* Treatment for Peyronie's Disease in a 70-Year-Old Patient

**DOI:** 10.1155/criu/2126627

**Published:** 2025-09-02

**Authors:** Matthew D. Wainstein, Sonia E. Masih, Timothy G. Schuster

**Affiliations:** ^1^Department of Urology, The University of Toledo College of Medicine and Health Sciences, Toledo, Ohio, USA; ^2^Genitourinary Surgeons, Promedica Toledo Hospital, Toledo, Ohio, USA

## Abstract

Collagenase *Clostridium histolyticum* (CCH) injections are the gold standard for nonsurgical management of Peyronie's disease. While generally well tolerated, penis fracture is a known complication. Cases typically occur during intercourse in the weeks following treatment. Because of this, patients are advised to abstain from sexual activity for 4 weeks postinjection. Here, we present a case of corporal rupture occurring 32 days after treatment. This is only the third reported case occurring 30 days after injection. While the risk of penis fracture likely decreases with time, these cases illustrate that the risk may extend beyond 4 weeks.

## 1. Introduction

Peyronie's disease (PD) is an acquired fibrotic disorder of the penis characterized by abnormal curvature. PD affects between 0.5% and 13% of men in the United States [1], with higher prevalence among those with comorbidities, such as diabetes, hypertension, hyperlipidemia, and smoking history [2–4]. The resulting curvature leads to deformity, penile shortening, pain, and erectile dysfunction (ED), as well as significant psychological distress related to sexual function and appearance [3–6]. PD progresses through an active and stable phase, distinguished by whether plaque formation and symptoms are ongoing or stabilized [7]. Management is guided by disease phase and degree of curvature [4, 7]. For patients with stable PD and penile curvature greater than 30°, intralesional collagenase *Clostridium histolyticum* (CCH), marketed as Xiaflex, is the gold standard and only FDA-approved pharmacologic therapy. Although CCH does not treat the pain or ED associated with PD, it has been shown to significantly decrease curvature and PD-specific psychosocial aspects [4, 8–10].

CCH injections are safe and generally well-tolerated. While postinjection penile ecchymosis, swelling, and pain are relatively common, these symptoms are typically mild and self-limiting [4, 8, 10]. A rare yet serious complication is corporal body rupture, or penile fracture [10]. The overall incidence is estimated to be around 0.6%, though smaller retrospective analyses have demonstrated rates as high as 4.9% [8, 10–12]. The risk is believed to be highest during the first 2 weeks after injection [13]. Initial product guidelines recommended abstaining from sexual intercourse for 2 weeks postinjection; however, this was later extended to 4 weeks following reports of fractures occurring beyond the initial period [13, 14]. While the risk is believed to decrease significantly after 4 weeks, the exact risk beyond this period remains uncertain. To date, there are only two reported cases of penile fracture occurring 30 days after injection [15, 16].

Here, we present a patient who experienced a penile fracture 32 days after CCH injections for treatment of PD.

## 2. Case Presentation

A 70-year-old male was referred to our clinic for evaluation and management of PD. He reported a spontaneous onset of dorsal penile curvature localized to the distal shaft. This occurred gradually over the course of 6 months without any preceding trauma or incident. He denied any pain with erections or decrease in penile tumescence; however, he could not have vaginal intercourse due to the curvature.

His past medical history was notable for hyperlipidemia, although he was not taking any daily medications. The patient did not have any prior history of ED. The patient denied any history of tobacco use but reported occasional alcohol consumption.

The initial degree of curvature was estimated from a photograph provided by the patient, which demonstrated approximately 40° of dorsal penile curvature. In-office, an artificial erection demonstrated a nontender, dorsally located, palpable plaque on the distal one-third of the shaft. After discussing treatment options, the patient elected to undergo CCH injections with penile remodeling.

The patient underwent one cycle of CCH treatment according to the manufacturer's instructions. This consisted of two occasions of in-office injections 3 days apart, followed by an in-office remodeling procedure 1 day after the final injection. The patient then continued regular at-home penile stretching and straightening over the course of the next 4 weeks per protocol. The patient reported some pain at the injection site, but otherwise no major complications were reported throughout this time.

Thirty-two days after the second injection, the patient resumed sexual activity while on vacation. This was in accordance with the 4 weeks of abstinence recommended by manufacturers. During intercourse, the patient reportedly felt a “pop” followed by immediate detumescence and massive penile swelling and ecchymosis. He presented to a local emergency room 1 day later and was evaluated by an urologist who diagnosed him with penile fracture without urethral injury. Ultimately, the patient chose to return home for further evaluation and management with our team.

The patient was seen in the clinic 2 days later. He reported significant swelling and bruising of his penis but denied any pain. On physical examination, there was extensive shaft ecchymosis and a palpable subcutaneous clot, consistent with penile fracture. He was subsequently brought to the operating room for exploration. A degloving circumcision incision revealed a sizable subcutaneous hematoma near the previous injection site. After being completely evacuated, a 1-cm defect in the tunica albuginea was repaired.

After 4 months, the patient reported partial improvement to his dorsal curvature but noted a new left-sided indent at the site of repair, which was causing difficulty with sexual intercourse. The treatment options were discussed, including further CCH injections versus penile plication. The patient elected for treatment with surgical plication initially but ultimately declined further intervention.

## 3. Discussion

Penile fracture is an uncommon genitourinary trauma, characterized by acute rupture of the tunica albuginea surrounding the corpus cavernosum. It most commonly occurs during erection, when the tunica is markedly thinned and under increased tension. Trauma to the penis most commonly occurs in the setting of sexual activity [10, 17, 18]. Penile fractures occurring after intralesional CCH therapy exhibit distinct features compared to traumatic penile fractures. Fractures occur almost exclusively at or in close proximity to the site of plaque injection and are thought to result from enzymatic weakening of the tunica combined with an applied external force [14, 17]. While sexual intercourse remains the most common precipitating factor, cases during spontaneous and nighttime tumescence have also been reported [10].

Due to the rarity of this complication, there are no current guidelines for management. Although surgical repair is typically indicated for traumatic penile fracture, CCH-associated ruptures are often managed conservatively [10]. Surgical repair in this context is technically challenging due to the compromised tissue integrity, as most ruptures occur in or near the site of injection [10]. Moreover, compromised tissue quality often necessitates grafting for successful repair [10, 14].

Several studies have shown comparable results for surgical and conservative management. In a survey of 100 physicians, Yafi et al. reported similar outcomes related to postfracture curvature, ED, and patient and physician satisfaction between surgical and conservative management [14]. Similarly, Kozacıoğlu et al. found that among 64 patients with penile fracture, worsening curvature occurred in 13% of cases treated surgically, while unchanged or improved curvatures occurred in 100% of cases treated without surgery [19]. While there are no definitive recommendations for the management of penile fracture in this context due to its low incidence, conservative management has been proposed as the primary choice unless there is evidence of urethral injury or an expanding hematoma [10]. Ultimately, treatment decisions should be made collaboratively with the patient after a thorough discussion of the potential risks and benefits.

Multiple reports have demonstrated that the risk of penile fracture after CCH is highest during the first 2 weeks posttreatment [13, 14]. While only a small number of fractures have been reported beyond 2 weeks, most either occurred prior to the guideline change or during sexual intercourse in violation of the recommended restriction [12, 13]. Notably, the two documented cases which occurred outside of 30 days were both associated with sexual intercourse, although detailed circumstances were not disclosed [15, 16]. These findings underscore sexual intercourse as the biggest risk for delayed corporal rupture and highlight the importance of adherence to postprocedure guidelines in mitigating late fracture risk.

Spontaneous erections also represent a significant risk factor for penile fracture following CCH injection [10, 13]. Although the majority of cases are reported within the first 2 weeks postinjection, Zucker et al. reported one case of spontaneous penile fracture occurring on Day 15 [13]. To date, there are no cases of spontaneous fracture beyond 30 days. These findings implicate spontaneous erection as an important nonmodifiable risk factor for penile fracture after CCH. However, it is important to recognize the potential for reporting bias due to the sensitive nature of the injury and the possibility of underreporting or misattribution of inciting events due to possible patient embarrassment.

Additional factors may contribute to the risk of penile fracture following CCH therapy, including degree of penile deformity prior to treatment and variations in penile traction or at-home remodeling. However, these factors are difficult to standardize and objectively assess. While comorbid conditions associated with PD may contribute to the risk of fracture, their impact is likely less significant than sexual intercourse and spontaneous erection. Furthermore, our patient did not exhibit any relevant comorbidities aside from hyperlipidemia. Further research to understand the mechanism leading to fracture may be necessary to determine additional risk factors.

This case highlights the importance of patient-centric counseling regarding posttreatment activity restrictions. While the overall risk is low, delayed corporal rupture can still occur despite adherence to the recommended abstinence guidelines.

## 4. Conclusion

Although penile fracture is being reported less often with the expansion of refraining from sexual activity from 2 to 4 weeks after CCH injections, it is still a known complication that men need to be counseled about when undertaking this treatment.

## Data Availability

Data sharing is not applicable to this article, as no new datasets were generated or analyzed during the current study.

## References

[1] Stuntz M., Perlaky A., des Vignes F., Kyriakides T., Glass D. (2016). The Prevalence of Peyronie’s Disease in the United States: A Population-Based Study. *PLoS One*.

[2] Usta M. F., Bivalacqua T. J., Jabren G. W. (2004). Relationship Between the Severity of Penile Curvature and the Presence of Comorbidities in Men With Peyronie’s Disease. *The Journal of Urology*.

[3] Hayat S., Brunckhorst O., Alnajjar H. M., Cakir O. O., Muneer A., Ahmed K. (2023). A Systematic Review of Non-surgical Management in Peyronie’s Disease. *International Journal of Impotence Research*.

[4] Nehra A., Alterowitz R., Culkin D. J. (2015). Peyronie’s Disease: AUA Guideline. *The Journal of Urology*.

[5] Smith J. F., Walsh T. J., Conti S. L., Turek P., Lue T. (2008). Risk Factors for Emotional and Relationship Problems in Peyronie’s Disease. *The Journal of Sexual Medicine*.

[6] Nelson C. J., Mulhall J. P. (2013). Psychological Impact of Peyronie’s Disease: A Review. *The Journal of Sexual Medicine*.

[7] Mulhall J. P., Schiff J., Guhring P. (2006). An Analysis of the Natural History of Peyronie’s Disease. *The Journal of Urology*.

[8] Gelbard M., Goldstein I., Hellstrom W. J. G. (2013). Clinical Efficacy, Safety and Tolerability of Collagenase Clostridium histolyticum for the Treatment of Peyronie Disease in 2 Large Double-Blind, Randomized, Placebo Controlled Phase 3 Studies. *The Journal of Urology*.

[9] Carson C. C., Sadeghi-Nejad H., Tursi J. P. (2015). Analysis of the Clinical Safety of Intralesional Injection of Collagenase *Clostridium histolyticum* (CCH) for Adults With Peyronie’s Disease (PD). *BJU International*.

[10] Hughes W. M., Natale C., Hellstrom W. J. G. (2021). The Management of Penile Fracture: A Review of the Literature With Special Consideration for Patients Undergoing Collagenase Clostridium histolyticum Injection Therapy. *Current Urology Reports*.

[11] Hellstrom W. J. G., Tue Nguyen H. M., Alzweri L. (2019). Intralesional Collagenase Clostridium histolyticum Causes Meaningful Improvement in Men With Peyronie’s Disease: Results of a Multi-Institutional Analysis. *The Journal of Urology*.

[12] Beilan J. A., Wallen J. J., Baumgarten A. S., Morgan K. N., Parker J. L., Carrion R. E. (2018). Intralesional Injection of Collagenase *Clostridium histolyticum* May Increase the Risk of Late-Onset Penile Fracture. *Sexual Medicine Reviews*.

[13] Zucker I., Nackeeran S., Mirza S., Masterson T. A. (2024). Risk Factors for Penile Fracture After Intralesional Collagenase Clostridium histolyticum in Peyronie’s Disease. *Urology*.

[14] Yafi F. A., Anaissie J., Zurawin J., Sikka S. C., Hellstrom W. J. G. (2016). Results of SMSNA Survey Regarding Complications Following Intralesional Injection Therapy With Collagenase Clostridium histolyticum for Peyronie’s Disease. *The Journal of Sexual Medicine*.

[15] Levine L. A., Cuzin B., Mark S. (2015). Clinical Safety and Effectiveness of Collagenase Clostridium histolyticum Injection in Patients With Peyronie’s Disease: A Phase 3 Open-Label Study. *The Journal of Sexual Medicine*.

[16] Yang K. K., Bennett N. (2016). Peyronie’s Disease and Injectable Collagenase Clostridium histolyticum: Safety, Efficacy, and Improvements in Subjective Symptoms. *Urology*.

[17] Pandher M., Fernandez Pedrosa G., Alwaal A. (2022). Presentation, Management, and Outcomes of Penile Fractures. *Journal of Men’s Health*.

[18] Falcone M., Garaffa G., Castiglione F., Ralph D. J. (2018). Current Management of Penile Fracture: An Up-to-Date Systematic Review. *Sexual Medicine Reviews*.

[19] Kozacıoğlu Z., Ceylan Y., Aydoğdu Ö., Bolat D., Günlüsoy B., Minareci S. (2017). An Update of Penile Fractures: Long-Term Significance of the Number of Hours Elapsed Till Surgical Repair on Long-Term Outcomes. *Turkish Journal of Urology*.

